# Supplementary material for: In silico analysis of local RNA secondary structure in influenza virus A, B and C finds evidence of widespread ordered stability but little evidence of significant covariation
Source: Sci Rep. 2022 Jan 10;12:310. doi: 10.1038/s41598-021-03767-x (PMC8748542; doi:10.1038/s41598-021-03767-x)
Supplement: Supplementary file 1 — Supplementary Information. [file 41598_2021_3767_MOESM1_ESM.zip › Supplemental Files/Supplemental File 3.zip!/Supplemental File 3/CM-BAll/pdf/IBV_seg5_2_1.R2R.sto.pdf]

IBV_seg5_2_1

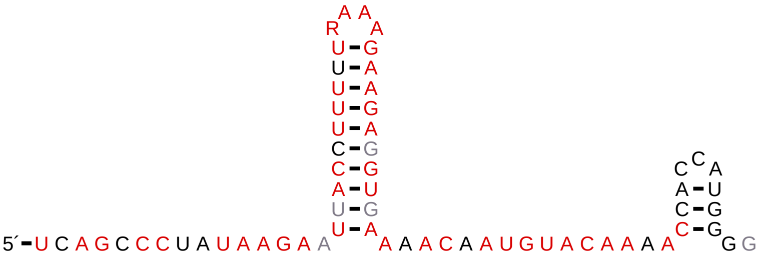

A A
R A
U G
U A
U A
U G
U A
C G
C G
A U
U G
U A
C
C A
A U
C G
C G
5´ U C A G C C C U A U A A G A A
A A A C A A U G U A C A A A A A
G G
